# Long non-coding RNA MALAT1 increases AKAP-9 expression by promoting SRPK1-catalyzed SRSF1 phosphorylation in colorectal cancer cells

**DOI:** 10.18632/oncotarget.7367

**Published:** 2016-02-13

**Authors:** Zhi-Yan Hu, Xiao-Yan Wang, Wen-bin Guo, Lin-Ying Xie, Yu-qi Huang, Yan-Ping Liu, Li-Wei Xiao, Sheng-Nan Li, Hui-Fang Zhu, Zu-Guo Li, Heping Kan

**Affiliations:** ^1^ Department of Pathology, Nanfang Hospital, Southern Medical University, Guangzhou, China; ^2^ Department of Pathology, School of Basic Medical Sciences, Southern Medical University, Guangzhou, China; ^3^ Guangdong Provincial Key Laboratory of Molecular Tumour Pathology, Southern Medical University, Guangzhou, China; ^4^ Department of Hepatobiliary Surgery, Nanfang Hospital, Southern Medical University, Guangzhou, China; ^5^ Department of Urology, Sun Yat-sen Memorial Hospital, Sun Yat-sen University, Guangzhou, China; ^6^ Department of Urology, The Third Affiliated Hospital of Southern Medical University, Southern Medical University, Guangzhou, China

**Keywords:** long non-coding RNA, MALAT-1, AKAP-9, SRSF1, colorectal cancer

## Abstract

Our earlier findings indicate that the long non-coding RNA MALAT1 promotes colorectal cancer (CRC) cell proliferation, invasion and metastasis *in vitro* and *in vivo* by increasing expression of AKAP-9. In the present study, we investigated the molecular mechanism by which MALAT1 enhances AKAP9 expression in CRC SW480 cells. We found that MALAT1 interacts with both SRPK1 and SRSF1. MALAT1 increases AKAP-9 expression by promoting SRPK1-catalyzed SRSF1 phosphorylation. Following MALAT1 knockdown, overexpression of SRPK1 was sufficient to restore SRSF1 phosphorylation and AKAP-9 expression to a level that promoted cell proliferation, invasion and migration *in vitro*. Conversely, SRPK1 knockdown after overexpression of MALAT1 in SW480 cells diminished SRSF1 phosphorylation and AKAP-9 expression and suppressed cell proliferation, invasion and migration *in vitro*. These findings suggest MALAT1 increases AKAP-9 expression by promoting SRPK1-catalyzed SRSF1 phosphorylation in CRC cells. These results reveal a novel molecular mechanism by which MALAT1 regulates AKAP-9 expression in CRC cells.

## INTRODUCTION

Long non-coding RNAs (LncRNAs) are arbitrarily defined as RNA molecules greater than 200 nucleotides in length that do not contain any apparent protein-coding potential, as determined largely through bioinformatics [1]. LncRNAs act by altering gene expression in a disease-, tissue- or developmental stage-specific manner [2]. Dysregulation of lncRNA expression has been linked to variety of human ailments, including Alzheimer's disease [3], ischemia [4], heart disease [5] and cancer [6]. Some LncRNAs play key roles in tumorigenesis. For instance, the lncRNA HULC is highly upregulated in liver cancer [7] and colon cancer-associated transcript-1 (CCAT1) is upregulated in gallbladder cancer [8].

An earlier study confirmed that metastasis associated lung adenocarcinoma transcript 1 (MALAT1), an 8.1-kb lncRNA transcribed from the nuclear-enriched transcript 2 (NEAT2), is upregulated in colorectal cancer (CRC) and that it promotes cell proliferation, migration and invasion *in vitro* by enhancing a kinase (PRKA) anchor protein 9 (AKAP-9) expression [9]. As a highly abundant nucleus-restricted RNA, MALAT1 interacts with serine-arginine splicing factors (SRSFs) and modulates their distribution to nuclear speckles. MALAT1 also regulates alternative splicing of pre-mRNAs by controlling the concentrations or phosphorylation of SRSFs [10–12]. SRSFs are a conserved family of proteins involved in alternative and constitutive splicing resulting in differential gene expression, and also play a part in mRNA export, genome stabilization, non-sense mediated decay, and translation [13–14]. Serine/threonine-protein kinase 1 (SRPK1) phosphorylates the SRSF family protein SRSF1, thereby regulating its assembly and localization [15]. In addition, SRPK1-catalyzed SRSF1 phosphorylation reportedly increases alternative splicing of tumor-related Rac1b in colorectal cells [16].

CRC is the third leading cause of cancer death in the world. Colorectal carcinogenesis is a multistep process involving progressive disruption of epithelial-cell proliferation, apoptosis, differentiation, and survival mechanisms [17–18]. The major cause of mortality in patients with colorectal tumors is metastasis [19], which is also a complex multistep and multigene process. Little is known about the key mechanisms and molecules involved in CRC invasion and metastasis.

Our earlier findings confirmed that the 6918 nt-8441 nt fragment located at the 3′ end of MALAT 1 plays a pivotal role in CRC metastasis [20]. They also verified the cancer-promoting activity of MALAT1 in CRC and identified AKAP-9 as a MALAT1-regulated gene [9]. This prompted us to test the idea that MALAT1 modulates AKAP-9 expression and function by promoting SRPK1-catalyzed SRSF1 phosphorylation in CRC cells.

## RESULTS

### MALAT1, SRPK1 and SRSF1 form a complex

**Figure 1:**
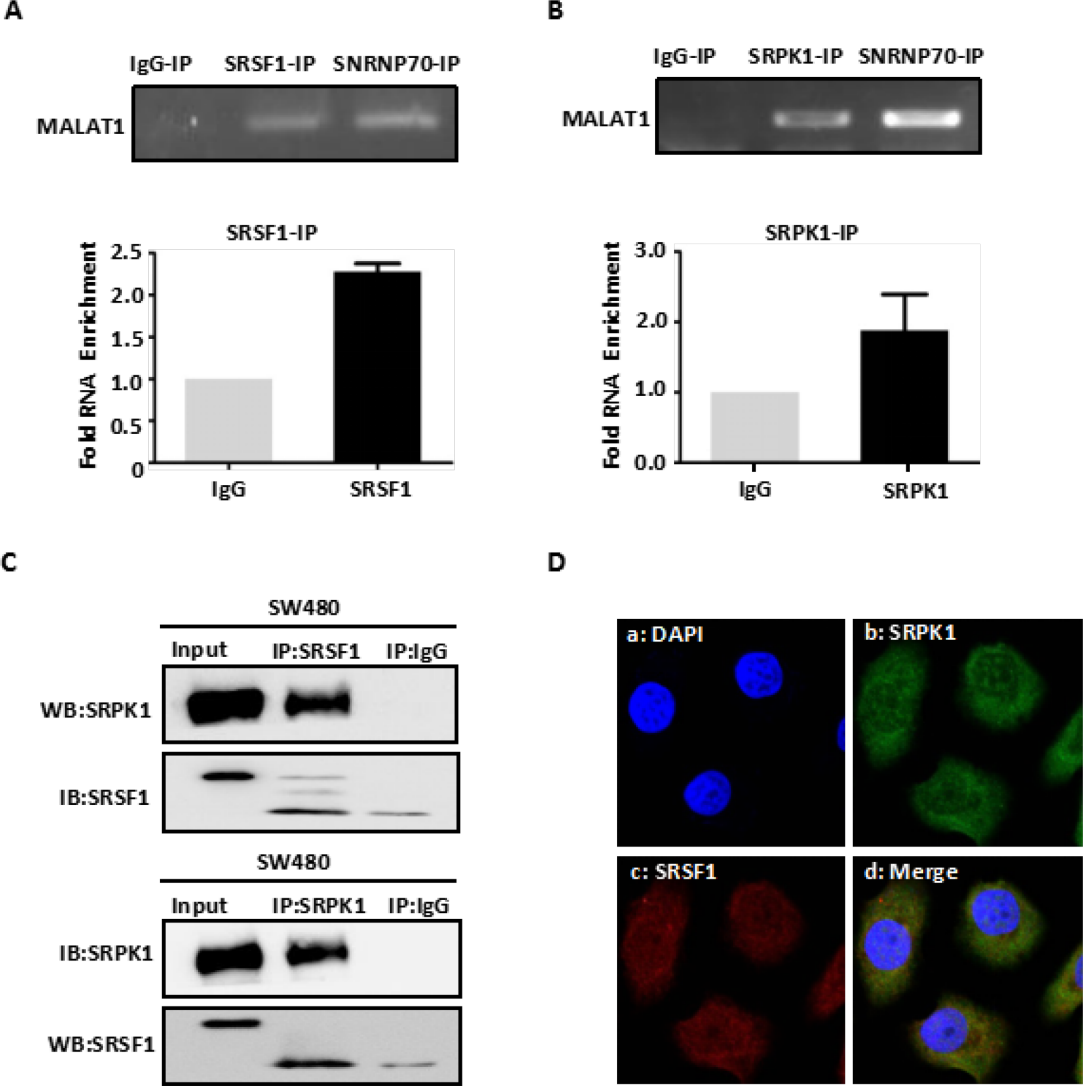
MALAT1 interacts with SRPK1 and SRSF1 protein (**A**) Immunoprecipitation from SW480 cells using SRSF1 antibody, mouse serum (negative control) or SNRNP70 (positive control), RT-PCR or qPCR from the IP samples using MALAT1-specific primers. Quantitative RT-PCR analysis of MALAT1 is expressed as fold enrichment over the IgG control. (**B**) Immunoprecipitation from SW480 cells using SRPK1 antibody mouse serum or SNRNP70 (positive control), RT-PCR or qPCR from the IP samples using MALAT1-specific primers. Quantitative RT-PCR analysis of MALAT1 is expressed as fold enrichment over the IgG control (**C**) SW480 cells were lysed and subjected to Co-IP with SRSF1 and SRPK1 antibody or mouse immunoglobulin G (IgG). Western blotting was performed to detect endogenous SRSF1 and SRPK1 in the precipitates (IP) or in cell lysates not subjected to IP (Input). (**D**) Intracellular localization of fluorescent-labeled SRPK1 and fluorescent-labeled SRSF1 by immunofluorescent staining. Immunofluorescent staining of SW480 cells treated with Alexa 488-labeled SRPK1 and Alexa 594-labeled SRSF1. Da: Nuclei were stained with DAPI (blue); Db: Alexa 488-labeled SRPK1 (green); Dc: Alexa 594-labeled SRSF1 (red); Dd: Superposition of Db and Dc demonstrated co-localization of SRPK1 and SRSF1 (yellow).

We previously found that MALAT1 promoted CRC growth and metastasis by regulating AKAP-9 gene expression [9]. In the present study, we used western blot and RT-PCR analyses to verified again that MALAT1 regulated AKAP-9 gene expression in SW480 CRC cells (Supplementary Figure 1A–1C). To explore the molecular mechanism by which MALAT1 affects AKAP-9 expression, we speculated that MALAT1 interacts with one or more splicing factors involved in AKAP-9 expression. This was confirmed by RNA co-immunoprecipitation (RNA-IP) assays using an anti-SRSF1 antibody in protein extracts from SW480 cells. RT-PCR and real-time PCR analyses of RNA-IP samples using primers for human MALAT1 revealed a specific interaction between MALAT1 and SRSF1 (Figure 1A). Because previous research showed that SRPK1 interacts with SRSF1 and enhances SRSF1 phosphorylation [15], we also used RNA-IP assays to test whether SRPK1 interacts with MALAT1 in SW480 cells. Our results show that SRPK1 does indeed interact with MALAT1 in SW480 cell (Figure 1B).

To investigate the specificity of the interaction between SRPK1 and SRSF1 in SW480 cells, we used immunofluorescent localization within SW480 cells and co-immunoprecipitation (Co-IP) from SW480 cell extracts with anti-SRPK1 and anti-SRSF1 antibodies, respectively. We found that SRPK1 specifically interacts with SRSF1 (Figure 1C–1D), which indicates that MALAT1, SRPK1 and SRSF1 likely form a complex.

### MALAT1 promotes SRSF1 phosphorylation by regulating SRPK1 expression and activity

**Figure 2:**
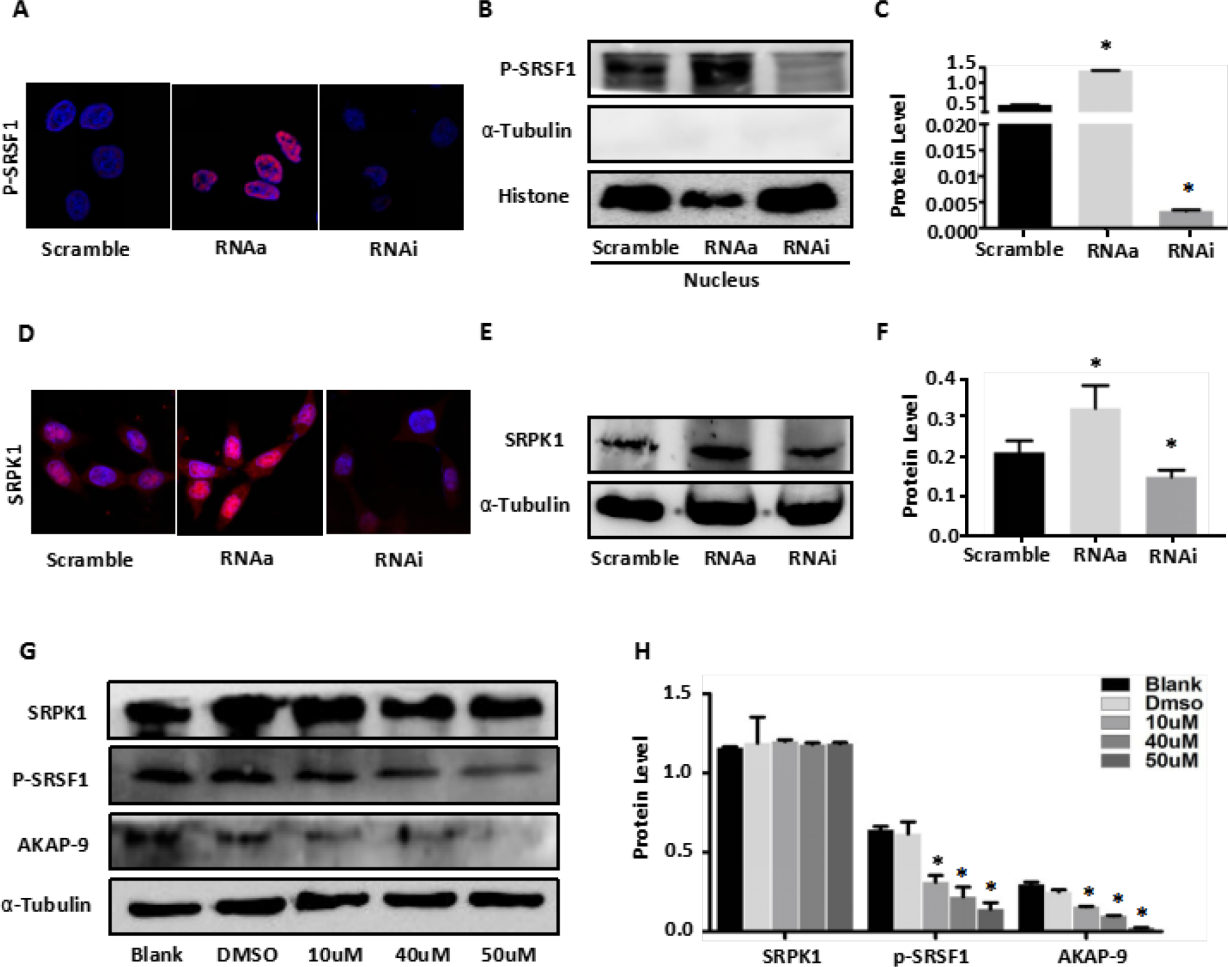
MALAT1 enhances AKAP-9 expression by promoting SRPK1-mediated SRSF1 protein phospholation (**A**) Immunostaining of phospholated SRSF1 protein in Scramble-SW480 RNAa-MALAT1-SW480 and RNAi-MALAT1-SW480 cells. Magnification: 1800x. (**B**) The phospholated SRSF1 protein in nucleus was detected by Western blot analysis using mAb1H4 antibody.(**C**) The phospholated SRSF1 protein levels were normalized to Histone. *P < 0.001 compared to the control group(Scramble), *n* = 3. (**D**) Immunostaining of SRPK1 protein in Scramble-SW480 RNAa-MALAT1-SW480 and RNAi-MALAT1-SW480 cells. Magnification: 1800x. (**E**) Western blot analysis of SRPK1 protein expression in Scramble-SW480 RNAa-MALAT1-SW480 and RNAi-MALAT1-SW480 cells. (**F**) The protein levels were normalized to α-Tubulin. *P < 0.05 compared to the control group (Scramble), *n* = 3. (**G**) RNAa-MALAT1-SW480 cells were treated with SRPK1 inhibitor SRPIN340 in different dose. (**H**) The protein levels were normalized to α-Tubulin. *P < 0.001 compared to the control group (Blank and dimethylsulphoxide 0.02%), *n* = 3.

To determine whether a MALAT1-SRPK1-SRSF1 complex functions within SW80 CRC cells, we assessed the levels of SRSF1 expression and phosphorylation in Scramble-SW480, RNAa-MALAT1-SW480 and RNAi-MALAT1-SW480 cells. We found that, by itself, MALAT1 had no significant effect on SRSF1 expression (Supplementary Figure 2A–2C). However, immunofluorescent detection revealed phosphorylated (phospho)-SRSF1 to be enriched in the nucleus, and activation of MALAT1 increased nuclear levels of phospho-SRSF1. Conversely, MALATI knockdown reduced levels of phospho-SRSF1 in SW480 cells (Figure 2A). As shown in Figure 2B–2C, similar results were obtained when nuclear phospho-SRSF1 levels were detected using western blotting. In addition, activation of MALAT1 increased SRPK1expression in SW480 cell, whereas MALAT1 knockdown decreased SRPK1 expression (Figure 2D–2F). These observations suggest that MALAT1 increases SRSF1 phosphorylation by stimulating SRPK1 expression.

To verify that changes in MALAT1-mediated SRSF1 phosphorylation was due to SRPK1 enzyme activity, RNAa-MALAT1-SW480 cells were treated with the SRPK1 inhibitor SRPIN340, which has been shown to block SRSF1 phosphorylation [30]. Western blot analysis of phospho-SRSF1 revealed that SRPIN340 (10 μM, 40 μM and 50μM for 48 h) dose-dependently inhibited SRSF1 phosphorylation induced by MALAT1 in RNAa-MALAT1-SW480 cells, as compared to control-treated cells (blank and dimethylsulphoxide 0.02%) (Figure 2G–2H). Moreover, SRPIN340 similarly inhibited MALAT1-medited AKAKP-9 expression in RNAa-MALAT1-SW480 cells, but had no effect on SRPK1 expression (Figure 2G–2H). These observations indicate that MALAT1 modulates its target gene AKAP-9 via promoting SRSF1 phosphorylation catalyzed SRPK1.

### Overexpression of SRPK1 restores AKAP-9 expression and activity by increasing phospho-SRSF1 levels in RNAi-MALAT1-SW480 cells

**Figure 3:**
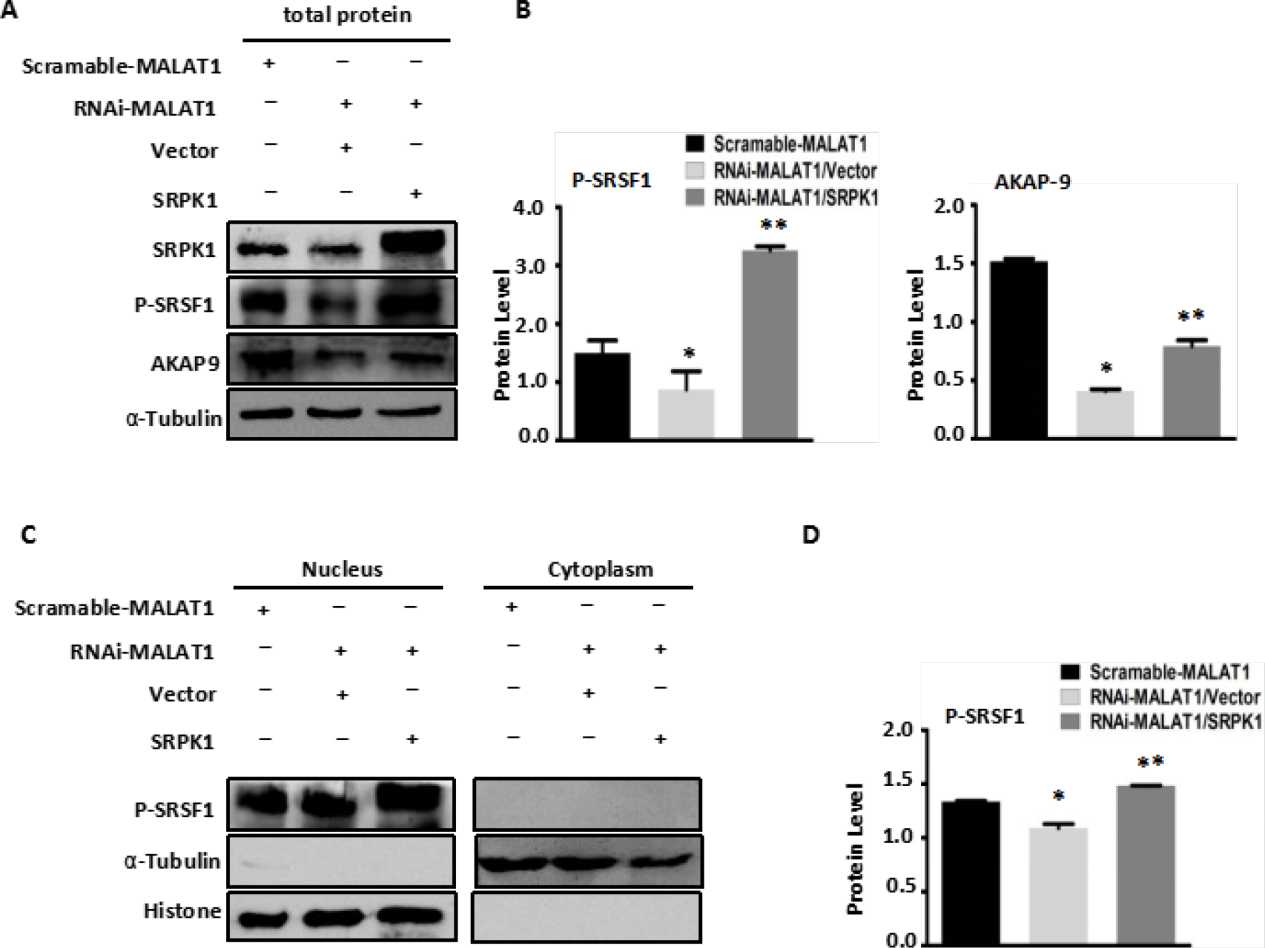
Overexpression of SRPK1 in MALAT1-deficient SW480 cells (RNAi-MALAT1) restored AKAP-9 expression by reversing the level of SRSF1 phosphorylation (**A**) Overexpression of SRPK1 in RNAi-MALAT1 SW480 cells restored phospholated SRSF1 and AKAP-9 protein expression. (**B**) The phospholated SRSF1 and AKAP-9 protein levels were normalized to α-Tubulin, *P < 0.05 compared to the control group (Scramle-MALAT1), **P < 0.01 compared to the control group (RNAi-MALAT1/Vector), *n* = 3. (**C**) The phospholated SRSF1 protein in nucleus and cytoplasm were detected by Western blot analysis using mAb1H4 antibody respectively. The phospholated SRSF1 protein is enriched in nucleus. (**D**) The phospholated SRSF1 protein levels were normalized to Histone. *P < 0.05 compared to the control group (Scramle-MALAT1), **P < 0.01 compared to the control group (RNAi-MALAT1/Vector), *n* = 3.

**Figure 4:**
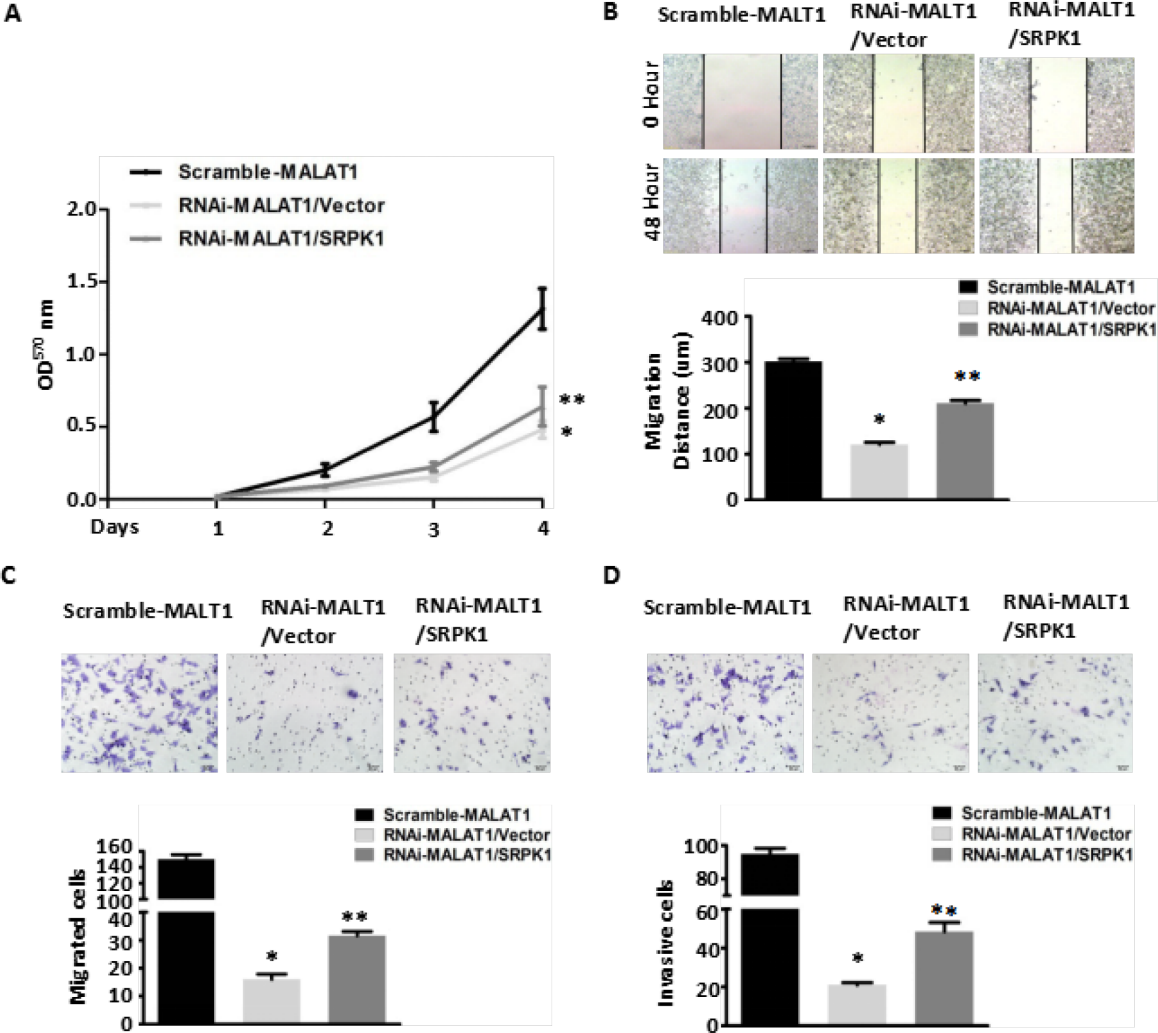
Overexpression of SRPK1 in MALAT1-deficient (RNAi-MALAT1) SW480 cells restored cell proliferation, migration and invasion inhibited by MALAT1 knockdown (**A**) Overexpression of SRPK1 in RNAi-MALAT1 SW480 cells restored cell proliferation inhibited by MALAT1 knockdown as measured by CCK8 assay. *P < 0.05 compared to the control group (Scramle-MALAT1); **P < 0.05 compared to the control group (RNAi-MALAT1/Vector) in Day (3) and Day (4), *n* = 3. (**B**–**C**) Cell migration was measured by both the wound-healing assay (B) and transwell migration assay (C). Overexpression of SRPK1 in RNAi-MALAT1 SW480 cells markedly restored the cells migration. *P < 0.001 compared to the control group (Scramle-MALAT1); **P < 0.05 compared to the control group (RNAi-MALAT1/Vector), *n* = 3. (**D**) Overexpression of SRPK1 in RNAi-MALAT1 SW480 cells restored cells invasion. The invasive capability was determined using matrigel invasion chambers. *P < 0.001 compared to the control group (Scramle-MALAT1); **P < 0.001 compared to the control group (RNAi-MALAT1/Vector), *n* = 3.

To confirm that MALAT1 modulates AKAP-9 expression by promoting SRPK1-catalyzed SRSF1 phosphorylation, we assessed the effect of SRPK1 overexpression on phospho-SRSF1 levels and AKAP-9 expression in RNAi-MALAT1-SW480 cells. Figure 3A–3B shows that overexpression of SRPK1 restored SRSF1 phosphorylation and AKAP-9 expression in RNAi-MALAT1-SW480 cells. We next examined the intracellular distribution of phospho-SRSF1. As shown in Figure 3C and 3D, SRPK1 overexpression led to increased levels of phospho-SRSF1 in the nucleus. Restoration of nuclear phospho-SRSF1 levels promoted alternative splicing of AKAP-9 PremRNA, thereby enhancing AKAP-9 protein expression. Notably, SRPK1 overexpression also markedly increased RNAi-MALAT1-SW480 cell proliferation (Figure 4A), migration (Figure 4B–4C) and invasion (Figure 4D).

### Down-regulation of SRPK1 expression decreases AKAP-9 expression and activity by reducing phospho-SRSF1 levels in RNAa-MALAT1-SW480 cells

**Figure 5:**
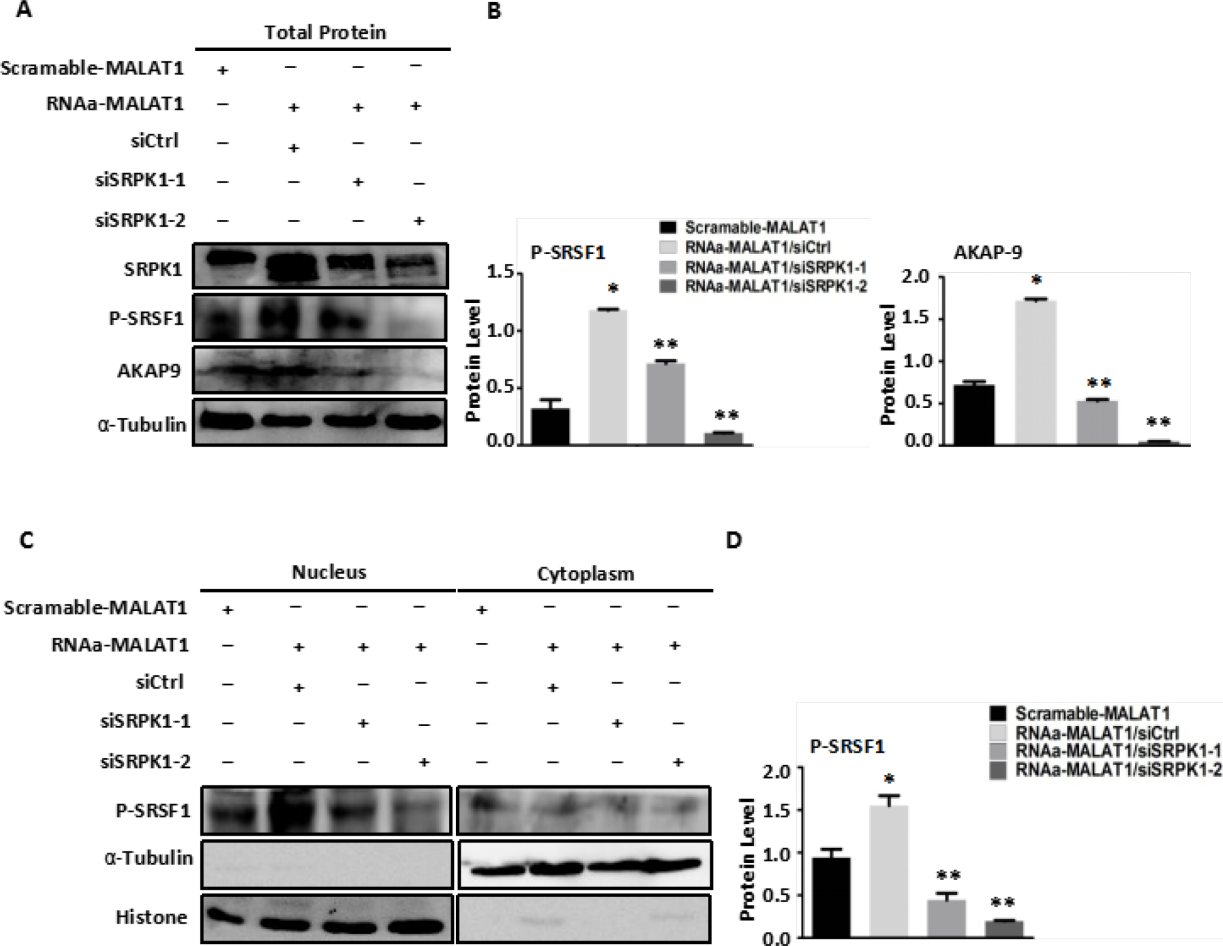
Knockdown of SRPK1 in MALAT1-activated SW480 cells (RNAa-MALAT1) attenuated AKAP-9 expression by attenuating the level of SRSF1 phosphorylation (**A**) Knockdown of SRPK1 in RNAa-MALAT1 SW480 cells attenuated phospholated SRSF1 and AKAP-9 protein expression. (**B**) The phospholated SRSF1 and AKAP-9 protein levels were normalized α-Tubulin. *P < 0.01 compared to the control group (Scramle-MALAT1), **P < 0.01 compared to the control group (RNAa-MALAT1/siCtrl), *n* = 3. (**C**) The phospholated SRSF1 protein in nucleus and cytoplasm were detected by Western blot analysis using mAb1H4 antibody respectively. The phospholated SRSF1 protein is enriched in nucleus. (**D**) The phospholated SRSF1 protein levels were normalized to Histone. *P < 0.05 compared to the control group (Scramle-MALAT1), **P < 0.01 compared to the control group (RNAa-MALAT1/siCtrl), *n* = 3.

**Figure 6:**
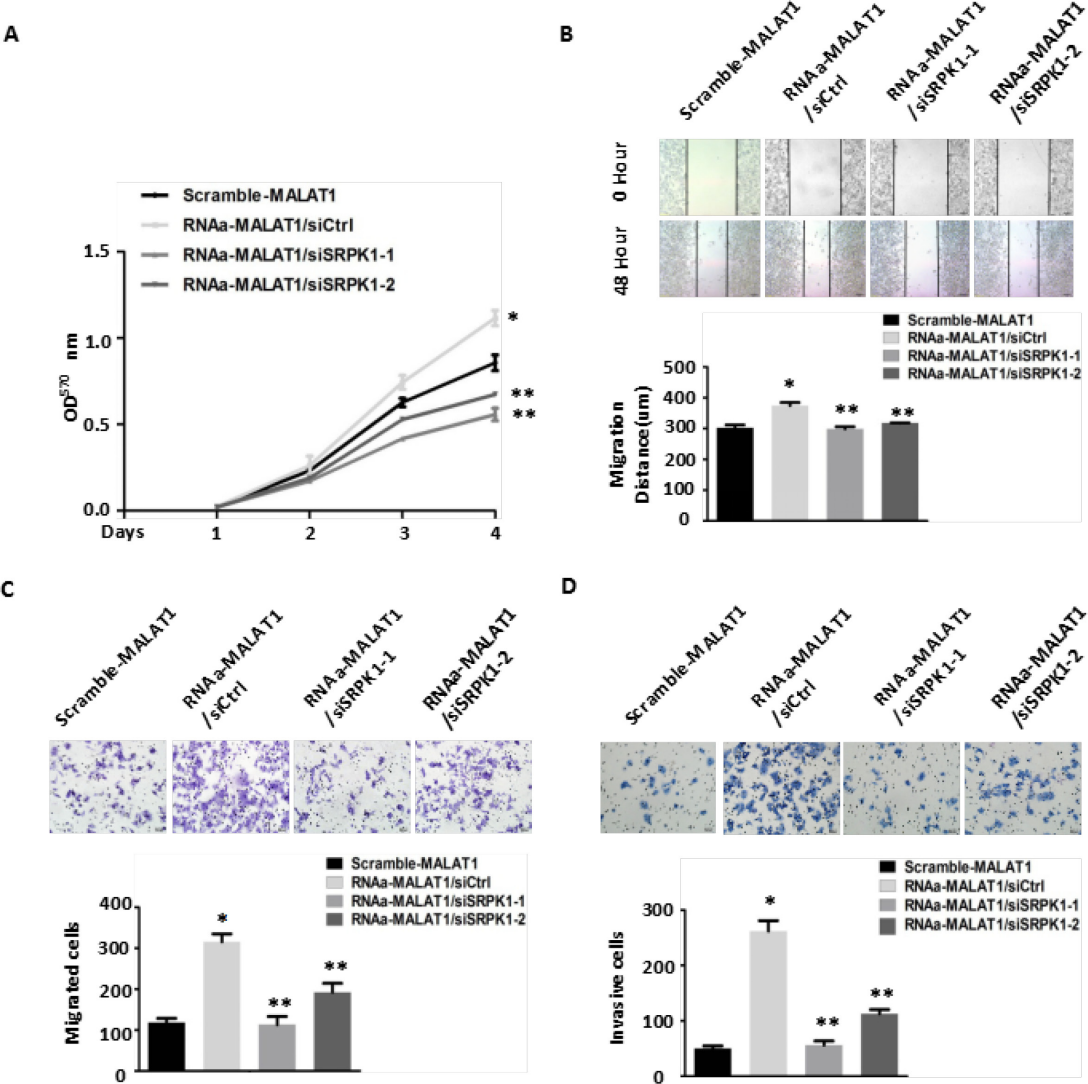
Knockdown of SRPK1 in MALAT1-activated (RNAa-MALAT1) SW480 cells attenuated cell proliferation, migration and invasion induced by MALAT1 overexpression (**A**) Knockdown of SRPK1 attenuated cell proliferation induced by MALAT1 overexpression as measured by CCK8 assay. *P < 0.05 compared to the control group (Scramle-MALAT1); **P < 0.05 compared to the control group (RNAa-MALAT1/siCtrl)) in Day (3) and Day (4), *n* = 3. (**B**–**C**) Cell migration was measured by both the wound-healing assay (B) and transwell migration assay (C). Knockdown of SRPK1 in RNAa-MALAT1 SW480 cells markedly attenuated the cells migration. *P < 0.01 compared to the control group (Scramble-MALAT1); **P < 0.01 compared to the control group (RNAa-MALAT1/siCtrl) in (B) and (C), *n* = 3. (**D**) Knockdown of SRPK1 in RNAa-MALAT1 SW480 cells inhibited cells invasion. The invasive capability was determined using matrigel invasion chambers. *P < 0.001 compared to the control group (Scramble-MALAT1); **P < 0.001 compared to the control group (RNAa-MALAT1/siCtrl), *n* = 3.

We examined the effect of down-regulated SRPK1 expression in RNAa-MALAT1-SW480 cells on AKAP-9 expression and SRSF1 phosphorylation. We found that decreasing SRPK1 expression led to reductions in phospho-SRSF1 levels, most notably in the nucleus, as well as reductions in AKAP-9 expression reflecting diminished alternative splicing of AKAP-9 PremRNA (Figure 5A–5D). The down-regulation of SRPK1 also attenuated the RNAa-MALAT1-SW480 cell proliferation (Figure 6A), migration (Figure 6B–6C) and invasion (Figure 6D) induced by activation of MALAT1. These data suggest that MALAT1-mediated SRPK1 expression leads to increases in the levels of SRSF1 phosphorylation and AKAP-9 expression, which in turn enhances cellular proliferation, migration and invasion.

## DISCUSSION

Recent evidence increasingly indicates that the lncRNA MALAT1 contributes to tumorigenesis in several types of human cancer, including colorectal [9], lung [21] and pancreatic cancers [22]. Our previous study showed vmetastasis by increasing expression of AKAP-9 [9]. In the present study, we found that MALAT1 localizes to nuclear speckles and interacts with several pre-mRNA splicing factors, including SRSF1, and that MALAT1 promotes SRSF1 phosphorylation within the cell nucleus [10].

SRSF1 is a member of the serine-arginine (SR) family of splicing proteins, which are well-recognized regulators of alternative splicing [23]. SR proteins regulate gene expression by acting at the post-transcriptional level to induce mRNA splicing [24], which alters the stability of the transcript [25] and its translation [26].

Research has long shown that SRPK1 catalyzes the phosphorylation of SRSF1, thereby activating it [27]. Our studies clearly show that MALAT1 and SRPK1 both interact with SRSF1 in SW480 CRC cells, which is consistent with earlier findings [27]. Within the resultant MALAT1-SRPK1-SRSF1 complex, MALAT1 plays not only a structural role, but also enhances SRSF1 phosphorylation by SRPK1. Interestingly, overexpression of MALAT1 in SW480 cells significantly increases SRPK1 expression and SRSF1 phosphorylation within the cell nucleus. This suggests SRPK1 activity promotes cellular proliferation and invasion [9], which is consistent the report that SRPK1 expression is higher in CRC tissues than in normal tissues [28]. In addition, by increasing the activation of SRSF1, and perhaps other SR splicing factors, MALAT1 enhances the alternative splicing of endogenous pre-mRNAs [10]. We therefore hypothesized that MALAT1 increases AKAP-9 expressionv by promoting SRPK1-catalyzed SRSF1 phosphorylation. It is possible that variation in AKAP9 expression is a consequence of changes in the distribution and/or ratio of phosphorylated and unphosphorylated SRSF1 pools. Consistent with that idea, we found that SRPK1 knockdown in MALAT1-activated SW480 cells significantly reduces MALAT1's ability to increase SRSF1 phosphorylation and AKAP9 expression, as well as in CRC cell proliferation, migration and invasion. Conversely, overexpression of SRPK1 in MALAT1-deficient SW480 cells significantly restores SRSF1 phosphorylation and AKAP9 expression. These observations suggest that MALAT1 increases AKAP9 expression by promoting nuclear SRSF1 phosphorylation catalyzed by SRPK1.

In summary, we have demonstrated that MALAT1 interacts with both SRPK1 and SRSF1 and that it promotes SRPK1 expression and SRSF1 phosphorylation in CRC cells, which in turn enhances AKAP-9 expression. Understanding the molecular mechanism by which MALAT1 promotes metastasis through increasing AKAP-9 expression may contribute to more effective and targeted therapy for CRC.

## MATERIALS AND METHODS

### Cell lines and culture

The stably-transduced cell lines RNAi-MALAT1-SW480 (RNA interference), RNAa-MALAT1-SW480(RNA activation), and Scramble-SW480 (control) were successfully established using lentiviral vector (pGCSIL-GFP, GeneChem) transduction of SW480 cells [9]. All CRC cells were cultured in RPMI 1640 medium (Gibco, USA) supplemented with 10% fetal bovine serum (FBS) (Gibco, USA) and maintained under standard conditions (5% CO^2^ and 95% atmosphere, 37°C).

### RNA coimmunoprecipitation

RNA-IP was performed using a Magna RIP™ RNA-binding protein immunoprecipitation kit according to the manufacturer's instructions (MILLIPORE). In brief, RNA immunoprecipitation was performed utilizing reversible chemical crosslinking of RNA-protein interactions by formaldehyde followed by immunoprecipitation using SRPK1 (Abcam) or SRSF1 antibodies (Abcam). After IP, extracts were reverse cross-linked, and total RNA was extracted using Trizol LS (Invitrogen, USA) and treated with RNase-free DNase I (Invitrogen, USA). MALAT1 expression was detected using both semi-quantitative polymerase chain reaction (PCR) and quantitative qPCR using PrimeScript^™^ PCR Master MixSYBR^®^ Premix Ex Taq^™^ (Tli RNaseH Plus) (TaKaRa, Code No: RR420A) with an ABI 7500 Real-Time PCR system. The primers for the real-time RT-PCR were designed with primer 5. The primer sequences for human MALAT1 were 5′-GGT AAC GAT GGT GTC GAG GTC-3′ (forward) and 5′-CCA GCA TTA CAG TTC TTG AAC ATG-3′ (reverse) The primer sequences for human glyceraldehyde-3 phosphate dehydrogenase (GAPDH) were 5′-ACA GTC AGC CGC ATC TTC TT-3′ (forward) and 5′-GAC AAG CTT CCC GTT CTC AG-3′ (reverse). GAPDH was used as an internal control that is comparable to a cyclophilin control. The assay was run in triplicate for each sample.

### Coimmunoprecipitation

Cells were washed twice with cold PBS and lysed in RIPA buffer (1 × PBS, 1% NP40, 0.1% SDS, 5 mM EDTA, 0.5% sodium deoxycholate, and 1 mM sodium orthovanadate) containing protease inhibitors at 4°C, then vortexed and centrifuged at 14,000 rpm for 10 min at 4°C. Total protein (500 μg) per sample was precleaned with 40 μL A-G beads (Santa Cruz Biotechnology) before immunoprecipitation with 2 μg control IgG (Santa Cruz Biotechnology), or SRPK1 (SRSF1) antibody (Abcam) at 4°C overnight. After incubation for 6 h with 40 μl of A-G beads at 4°C followed by 5 washes with PBS containing 0.2% NP-40, proteins bound to the A-G beads were released into 2 × SDS-PAGE sample buffer by boiling for 5 min and used for Western blotting with anti-SRSF1 for SRPK1 and anti-SRPK1 for SRSF1 (Abcam).

### RNA extraction, RT-PCR and real-time RT-PCR

Total RNA was isolated from the cells using the TRIzol procedure (Takara, Code No.108). Ten micrograms of RNA from each sample were added to 20 ml of reaction mixture, and cDNA was synthesized using a PrimeScript^™^ RT reagent Kit with gDNA Eraser (TaKaRa, Code No. R047A). Amplification of a GAPDH fragment was used as an internal quantitative control. Real-time RT-PCR with SYBR^®^ Premix Ex Taq^™^ (Tli RNaseH Plus) (TaKaRa, Code No. R420A) was used to calculate mRNA expression. The primers for the real-time RT-PCR were designed with primer 5. The primer sequences used were as follows: for human MALAT1, 5′-GGT AAC GAT GGT GTC GAG GTC-3′ (forward) and 5′-CCA GCA TTA CAG TTC TTG AAC ATG-3′ (reverse); for human AKAP-9, 5′-ACT CAA GGC ACA GCA TAA ACA C -3′ (forward) and 5′-GTT CTT CAC TGC GTC CCA A -3′ (reverse); for human SRSF1, 5′-GGA ACA ACG ATT GCC GCA TCT A-3′ (forward) and 5′-CTT GAG GTC GGA TGT CGC GGA TA-3′ (reverse); and for human GAPDH, 5′-ACA GTC AGC CGC ATC TTC TT-3′ (forward) and 5′-GAC AAG CTT CCC GTT CTC AG-3′ (reverse). The PCR protocol entailed incubation at 95°C for 10 min, followed by 40 cycles of denaturing at 95°C for 10 s, annealing at 60°C for 30 s and extension at 72°C for 30 s, followed by a final extension at 95°C for 15 s. Expression data were normalized to the geometric mean of GAPDH to control for variability in the expression levels, which were analyzed using the 2-ΔΔCt method to determine relative gene expression.

### Lentivirus preparation

MALAT1 gene expression was knocked down using RNA interference (RNAi) targeting on MALAT1 mRNA. The interference sequence the MALAT1 shRNA was 5′-CACCGGGCAAATATTGGCAATTAGTCCGAAGAC TAATTGCCAATATTTGCCC-3′. The RNAi cDNA sequence was cloned into the pGCSIL-GFP lentiviral expression vector according to the manufacturer's instructions (GeneChem). According to principles of Watson-Crick base pairing, saRNAs react with the promoter and GC rich region of mRNAs to trigger endogenous gene expression. MALAT1 gene was overexpressed by RNA activation (RNAa) targeting the promoter region of MALAT1 gene, which was analyzed for promoter motifs and high GC domains using Promoter Scan Searcher and CpG Island Searcher software. The promoter-dsDNA sequence targeting 592–574 bp of the MALAT1 gene sequence were 5′-CGAGAAUU CUAGACUAGUATT-3′ (forward) and 3′-UACUAGUCUAGAAUUCUCGTT-5′ (reverse) The promoter-dsDNA sequence was also cloned into the pGCSIL-GFP lentiviral expression vector according to the manufacturer's instructions (GeneChem) [9].

### Transfection of plasmids and siRNAs

Plasmids harboring siRNAs were transfected into cells using Lipofectamine 2,000 (Invitrogen) according to the manufacturer's instructions. Full-length human wild-type SRPK1 cDNA was inserted into CMV-MCS-3FLAG-SV40-Neomycin expression vector from Shanghai Genechem Co. All constructs were verified for sequence correctness by direct sequencing. The sequences of two siRNAs against SRPK1 were synthesized by Shanghai GenePharma Co. The sense sequences for the two siRNAs against SRPK1 were #1, GGG UCU UGA UUA UUU ACA UTT and #2, GCC AGG AUC AAA CGC UUA UTT. Efficacies of the plasmids and siRNAs were verified by western blotting of cellular SRPK1.

### Western blotting

Cell lysates were prepared using SDS lysis solution. Protein concentrations were measured using a BCA protein assay kit. Equal amounts of protein were separated by electrophoresis on 10% SDS-polyacrylamide gel and then electrotransferred from the gel to nitrocellulose membranes. The membranes were blocked with 5% non-fat milk solution for 1 h and then incubated with primary monoclonal antibodies against AKAP-9 (Abcam), SRPK1 (Abcam) and SRSF1 (Abcam) overnight at 4°C. Phospho-SRSF1 was incubated with mAb1H4 antibody [29]. α-Tubulin was used as an internal control. After washing with TBS-T, the membrane was incubated with secondary antibodies against goat or mouse IgG. The membrane was then washed, and blots were detected using an enhanced chemiluminescence (ECL) detection system (Thermo) according to the manufacturer's instructions.

### Cell immunofluorescence

Cells plated on poly-L-lysine-coated glass coverslips were fixed with 4% paraformaldehyde and washed with PBS, after which they were permeabilized in 0.1% Triton X-100/PBS for 10 min. The cells were then incubated with primary antibodies followed fluorescein isothiocyanate-tagged secondary antibodies. 4′,6-Diamidino-2-phenylindole (DAPI) served as a nuclear counterstain. The fluorescence was recorded using an inverted fluorescence microscope (Leica, Germany).

### Proliferation, cell migration, and invasion assays

Proliferation, colony formation, migration, and invasion by transfected CRC cells were determined as previously described [9].

### Statistical analysis

All statistical analyses were performed using the SPSS 16.0 (Abbott Laboratories, USA). All results were confirmed by statisticians in the Department of Health Statistics, Southern Medical University. Quantitative values are expressed as the mean ± SD. Differences among/between sample groups were analyzed using one-way ANOVA or independent samples *t* tests.

## SUPPLEMENTARY MATERIALS FIGURES


