# Supplementary material for: Long non-coding RNA MALAT1 increases AKAP-9 expression by promoting SRPK1-catalyzed SRSF1 phosphorylation in colorectal cancer cells
Source: Oncotarget. 2016 Feb 13;7(10):11733–43. doi: 10.18632/oncotarget.7367 (PMC4905507; doi:10.18632/oncotarget.7367)
Supplement: Supplementary file 1 [file oncotarget-07-11733-s001.pdf]

Long non-coding RNA MALAT1 increases AKAP-9 expression by promoting SRPK1-catalyzed SRSF1 phosphorylation in colorectal cancer cells

Supplementary Materials

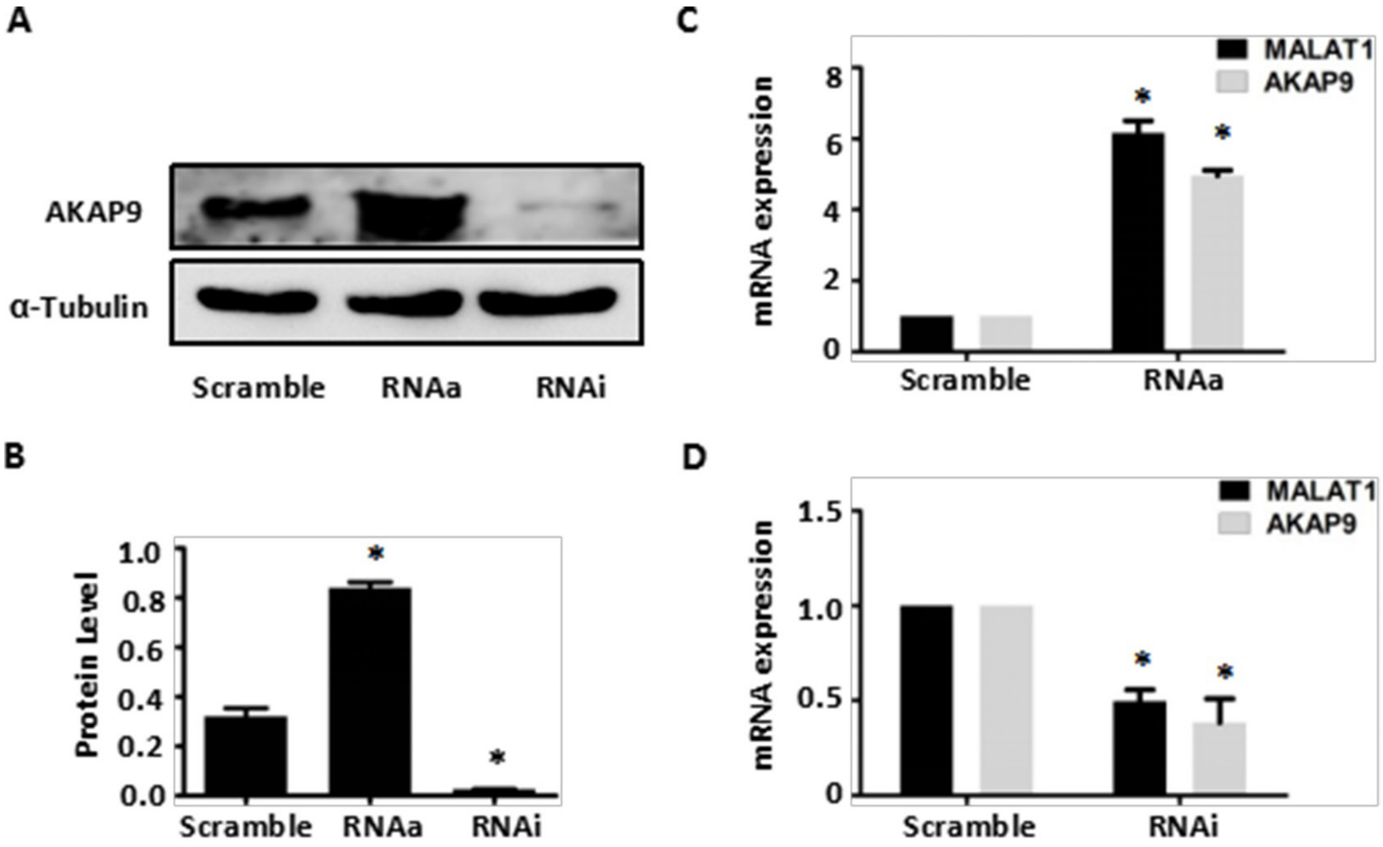

Supplementary Figure S1: Long non-coding RNA MALAT1 regulated AKAP-9 expression in CRC SW480 cells. (**A**) Western blot analysis of AKAP-9 protein expression in SW480 cells with stably activated\down-expressing MALAT1 (RNAa\RNAi) or scramble. (**B**) The protein levels were normalized to α-Tubulin. *$P < 0.001$ compared to the control group (scramble), $n = 3$. (**C–D**) AKAP-9 mRNA expression were detected by qRCR. *$P < 0.01$ compared to the control group (scramble), $n = 3$.

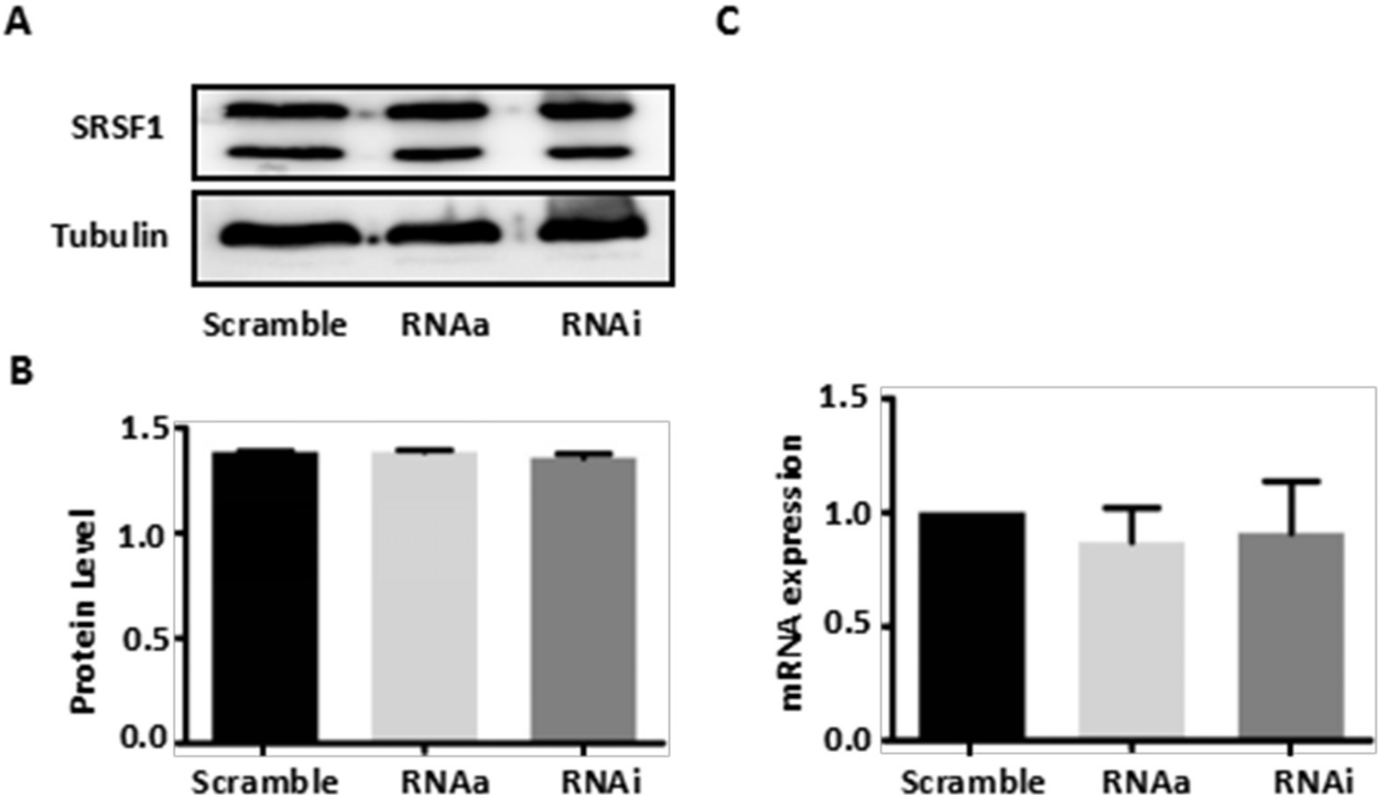

Supplementary Figure S2: Long non-coding RNA MALAT1 did not regulate SRSF1 protein expression in CRC SW480 cell. (**A**) Western blot analysis of SRSF1 protein expression in SW480 cells with stably activated\down-expressing MALAT1 (RNAa\RNAi) or scramble. (**B**) The protein levels were normalized to α-Tubulin, *n* = 3. (**C**) SRSF1 mRNA expression were detected by qRCR, *n* = 3.
